# Characteristics, treatment and survival in de novo and metachronous metastatic breast cancer: a nationwide comparative analysis

**DOI:** 10.1007/s10549-026-07979-5

**Published:** 2026-05-12

**Authors:** Ellis Slotman, Linda de Munck, Agnes Jager, Aafke H. Honkoop, Ester J. M. Siemerink, Joan B. Heijns, Elsken van der Wall, Natasja J. H. Raijmakers, Heidi P. Fransen, Sabine Siesling

**Affiliations:** 1 Departement of Health Technology and Services Research (HTSR), Technical Medical Centre, University of Twente, Enschede, The Netherlands; 2 Department of Research and Development, Netherlands Comprehensive Cancer Organisation (IKNL), Utrecht, The Netherlands; 3 Department of Medical Oncology, Erasmus MC Cancer Institute, Rotterdam, The Netherlands; 4 Department of Medical Oncology, Isala Clinics, Zwolle, The Netherlands; 5 Department of Medical Oncology, Ziekenhuisgroep Twente, Hengelo, Netherlands; 6 Department of Medical Oncology, Amphia Hospital, Breda, The Netherlands; 7 Department of Medical Oncology, University Medical Center Utrecht, Utrecht University, Utrecht, The Netherlands

**Keywords:** Metastatic breast cancer, De novo, Metachronous, Characteristics, Treatment, Survival

## Abstract

**Purpose:**

To compare patient characteristics, treatment patterns, and survival between de novo and metachronous metastatic breast cancer (MBC) using nationwide data.

**Methods:**

A total of 2,366 MBC patients (900 de novo, 1,466 metachronous) diagnosed in 2019 were selected from the Netherlands Cancer Registry. Patient- and tumor characteristics and systemic treatment patterns were compared using chi-squared or Fisher’s exact tests. Overall survival (OS) was compared using Kaplan-Meier curves and Cox proportional hazard analyses. All analyses were stratified by clinical subtype (HR+/HER2-, HR+/HER2+, HR-/HER2+, HR-/HER2-). For patients with HR+/HER2 − tumors, a sub-analysis examined OS in de novo versus metachronous MBC, stratifying the latter by receipt of prior (neo)adjuvant systemic treatment.

**Results:**

De novo MBC patients were younger, had more HER2-positive (22% vs. 11%) and fewer triple-negative tumors (11% vs. 16%). Patients with metachronous MBC more often had CNS metastases and metastases in other localizations than the lymph nodes, bone, visceral organs and CNS. Among HER2 + patients, chemotherapy and targeted therapy were more often administered in de novo versus metachronous MBC. Median OS was longer in de novo MBC for HR+/HER2- tumors (40.8 vs. 30.3 months, aHR 1.27, 95%CI 1.12–1.43) and HR−/HER2 + tumors (51.1 vs. 9.1 months, aHR 1.62, 95%CI 1.03–2.54). In HR+/HER2 − patients, metachronous MBC patients who received prior (neo)adjuvant systemic treatment had worse OS than de novo cases (prior chemotherapy: aHR 1.52, 95%CI 1.29–1.78); prior hormonal therapy only: aHR 1.33, 95%CI 1.10–1.61), whereas those without prior systemic treatment had similar outcomes.

**Conclusion:**

De novo and metachronous MBC have different tumor biology, treatment patterns, and survival. In metachronous MBC patients, prior (neo)adjuvant systemic treatment was associated with worse survival compared to de novo MBC or patients with metachronous MBC without prior (neo)adjuvant treatment.

**Supplementary Information:**

The online version contains supplementary material available at 10.1007/s10549-026-07979-5.

## Introduction

With over 2 million new cases of invasive breast cancer diagnosed each year, breast cancer remains the most common cancer diagnosed in women worldwide [1]. In the Netherlands, approximately 5% of breast cancer patients are diagnosed with de novo metastatic breast cancer (MBC), defined as distant metastases present at the initial diagnosis [2]. The proportion of patients developing metastatic disease after diagnosis and treatment of non-metastatic breast cancer, termed metachronous MBC, ranges from approximately 2–8% in patients with early stage disease to approximately 25–40% in patients with locally advanced disease [3–5].

Previous studies have reported differences in characteristics and survival between patients with de novo and metachronous MBC. In general, patients with de novo MBC present with more favorable disease characteristics and experience longer overall survival compared to patients with metachronous MBC [3, 6–8]. However, much of the existing evidence is based on small cohorts or population-based data that compare patients with de novo and metachronous MBC diagnosed in different time periods. Since treatments develop and change over time, this may introduce bias. Moreover, while previous studies have identified differences in clinical subtypes between de novo and metachronous MBC, most available comparisons of patient characteristics and outcomes between de novo and metachronous MBC did not stratify by subtype. This is a critical gap, since disease characteristics, treatment strategies and prognosis inherently depend on clinical subtype. Therefore, the differences observed between de novo and metachronous MBC in earlier research may be attributable to underlying subtype distributions, rather than to the timing of metastatic disease itself. Additionally, patients with metachronous MBC may have received prior treatment for early-stage disease, potentially affecting treatment options and response due to residual toxicities or resistance.

To address the limitations of previous studies and provide a more accurate comparison, our study uses a nationwide cohort of patients diagnosed with either de novo or metachronous metastatic disease in 2019. We aimed to compare clinicopathological characteristics, treatment patterns, and survival outcomes between de novo and metachronous MBC, with stratification by clinical subtype and consideration of prior treatment exposure to better understand the effect of disease timing in MBC.

## Methods

### Patient selection

In this study, all patients diagnosed in 2019 with either de novo or metachronous MBC were selected from the Netherlands Cancer Registry (NCR). The NCR is a nationwide cancer registry hosted by the Netherlands Comprehensive Cancer Organisation (IKNL) and includes data on all patients newly diagnosed with cancer based on notification by the Nationwide automated pathology archive (PALGA) since 1989 [9]. All cases of de novo metastases are identified and recorded routinely in the NCR. De novo MBC cases in this study included all patients diagnosed with metastases within 30 days of primary tumor diagnosis to capture those patients in whom metastases were present at initial diagnosis. The Dutch oncology norms specify completing diagnostic work‑up within three weeks and initiating treatment within six weeks of first consultation [10]. Therefore, a 30‑day window reflects the period in which metastases present at diagnosis would typically be detected, while excluding those identified only after treatment initiation. Metachronous metastatic breast cancers are not registered routinely, but were available in the NCR for diagnosis year 2019 on a nationwide scale due to notification by means of one or more of the following pathways: (1) metachronous metastatic breast cancer cases diagnosed in 2019 that were registered previously in the NCR through specific registration projects (i.e., cohorts of patients with non-metastatic breast cancer diagnoses from specific incidence years or included in specific projects that have been manually checked by the NCR data managers for the occurrence of metachronous metastatic breast cancer), (2) metachronous metastatic breast cancer cases notified through the National Pathology Archive (PALGA), (3) metachronous metastatic breast cancer cases identified by using an algorithm applied to data from the Dutch National Hospital Care Registration (LBZ). The LBZ contains medical data (diagnostic codes, medical procedures, dispensed medications) of all patients who visited a Dutch hospital or had a digital contact moment. The algorithm calculated a probability (0-100%) of having developed metachronous metastases for patients with a previous diagnosis of non-metastatic breast cancer in the NCR. The probability was calculated using features in the LBZ (medical procedures, medication and diagnostic codes). The presence of metachronous metastases was then confirmed by NCR data managers by review of the medical records of all patients with a probability value of ≥ 60%, and additional data on date of diagnosis, localization of the metastases and treatment of these cases was collected.

### Data and definitions

For all included patients, data on patient characteristics (age at diagnosis of metastasis and socio economic status at primary tumor diagnosis), primary tumor characteristics (morphology, differentiation grade, receptor status), localization of metastases, receptor status of metachronous metastases and systemic treatment (both after primary tumor diagnosis and in the metastatic setting) were used in this study.

Metastatis free interval was determined for patients with metachronous metastases based on the interval between the primary tumor diagnosis and diagnosis of first metachronous metastasis and was categorized as < 1 year, 1–5 years or > 5 years. Socio-economic status was determined at primary tumor diagnosis and categorized into low, medium, or high based on the median household income of a 6 digit postal code area. Clinical subtype was categorized into HR+/HER2+, HR+/HER2-, HR-/HER2+, or HR-/HER2-. This categorization was based on the receptor status of the primary tumor for patients with de novo metastatic disease, and on the receptor status of the metachronous metastasis for those with metachronous metastatic disease. In case the receptor status of the metachronous metastasis was unknown, clinical subtype was based on the receptor status of the primary tumor when available. Following this substitution, most patients with missing data at the time of metachronous metastasis were classified as either HR+/HER2- or HR-/HER2- based on primary tumor data (Supplementary Table 1). Among patients whose receptor status was known at both time points, these subtypes showed concordance in more than 80% of cases (Supplementary Table 2), suggesting that this approach provides a reasonably accurate classification in the majority of cases.

All data on patient and tumor characteristics, and data on (neo)adjuvant systemic treatment for the primary non-metastatic breast tumor were collected from the NCR. Data on systemic treatment in the metastatic setting for both de novo and metachronous metastatic disease were obtained from medical procedures and dispensed medications that were registered in hospital records and included in the LBZ. All systemic treatments received within the first year after diagnosis of metastatic disease were included in this study. Information on vital status in the NCR is available by annual linkage of the NCR to the Dutch Personal Records Database and was updated until February 1, 2025.

### Statistical analyses

Clinicopathological characteristics and treatment patterns were analyzed using descriptive statistics and compared between patients with de novo and metachronous MBC using chi-squared tests or Fisher exact tests. Overall survival (OS) was assessed from primary diagnosis (de novo metastatic disease) or date of first metastasis (metachronous metastatic disease) until death or end of follow-up. OS was compared using Kaplan-Meier survival curves, log rank tests and Cox regression analyses. To evaluate the effect of prior systemic treatment for non-metastatic breast cancer on survival in patients with metachronous MBC, additional analyses were performed in the subgroup of patients with HR+/HER2 − MBC who received systemic treatment in the metastatic setting. This subgroup was selected because only in HR+/HER2 − MBC were there sufficient numbers of patients without prior systemic treatment to allow for meaningful stratification. In this subgroup survival was compared across four groups: (1) patients with de novo MBC, (2) patients with metachronous MBC who received prior chemotherapy (with or without hormonal therapy), (3) patients with metachronous MBC who received prior hormonal therapy only, and (4) patients with metachronous MBC who did not receive prior systemic treatment.

## Results

### Clinicopathological characteristics

A total of 2,366 patients diagnosed with metastatic breast cancer in the Netherlands in 2019 were included, including 900 patients with de novo MBC and 1,466 patients with metachronous MBC (Table 1). Patients with de novo MBC were more often younger than 50 years (20% vs. 12%), whereas patients with metachronous MBC were more often between the ages of 50–70 (52% vs. 44%). Patients with de novo MBC had slightly more often a low socioeconomic status compared to patients with metachronous MBC (25% vs. 21%). HER2 + tumors were more often observed in de novo versus metachronous MBC (22% vs. 11%, *p* < 0.001). Triple negative tumors (HR- / HER2-) were less often observed in de novo MBC (11% vs. 16%).

In metachronous metastatic disease, a metastasis-free interval > 5 years was more common in hormone receptor positive tumors (HR+ / HER2+: 46%, HR+ / HER2-: 59%) compared to hormone receptor negative tumors (HR- / HER2+: 23%, HR- / HER2-: 20%) (Table 2). Across most clinical subtypes, patients with metachronous MBC more often had central nervous system (CNS) metastases as well as metastases in other localizations than the lymph nodes, bone, visceral organs and CNS. In patients with HER2- tumors, bone-only metastases were less frequently observed in metachronous MBC versus de novo MBC (HR+ / HER2-: 28% vs. 40% *p* < 0.001, HR- / HER2-: 7% vs. 18% *p* = 0.003), while lung metastases were observed more frequently (HR+ / HER2-: 26% vs. 20% *p* = 0.004, HR- / HER2-: 40% vs. 28% *p* = 0.04).

**Table 1:** Patient characteristics and clinical subtype of patients diagnosed with de novo or metachronous metastatic breast cancer (MBC) in 2019

	De novo MBC	Metachronous MBC	*P*-value^a^
	*N* (%)	*N*(%)	
N	900	1466	
**Patient characteristics**			
Age at diagnosis of metastases			
< 50	183 (20)	177 (12)	< 0.001
50–70	392 (44)	763 (52)	
70+	325 (36)	526 (36)	
Socio-economic status at primary cancer diagnosis			
Low	225 (25)	248 (21)	0.004
Middle	478 (53)	621 (52)	
High	195 (22)	329 (27)	
Unknown	2	268	
ER status			
ER-	195 (22)	302 (21)	0.54
ER+	686 (78)	1132 (79)	
Unknown/not determined	19	32	
PR status			
PR-	373 (42)	651 (46)	0.13
PR+	506 (58)	774 (54)	
Unknown/not determined	21	41	
HER2 status			
HER2-	667 (78)	1221 (89)	< 0.001
HER2+	190 (22)	156 (11)	
Unknown/not determined	43	89	
Clinical subtype			
HR+/HER2+	101 (12)	109 (8)	< 0.001
HR+/ HER2-	571 (67)	995 (72)	
HR-/ HER2+	89 (10)	47 (3)	
HR- / HER2-	94 (11)	225 (16)	
Unknown/not determined	45	90	

^a^ P-values were calculated over known values only

**Table 2:** Disease characteristics by clinical subtype of patients diagnosed with de novo or metachronous metastatic breast cancer (MBC) in 2019

Clinical subtype^a^	HR+ / HER2+		*P-value* ^b^	HR+ / HER2-		*P-value* ^b^	HR- / HER2+		*P-value* ^b^	HR- / HER2-		
	De novo MBC	Metachronous MBC		De novo MBC	Metachronous MBC		De novo MBC	Metachronous MBC		De novo MBC	Metachronous MBC	*P-value* ^b^
	*N*(%)	*N* (%)		*N*(%)	*N* (%)		*N*(%)	*N* (%)		*N*(%)	*N* (%)	
**N**	101	109		571	995		89	47		94	225	
Metastasis-free interval												
0 years (synchronous MBC)	101 (100)	NA	NA	571 (100)	NA	NA	89 (100)	NA	NA	94 (100)	NA	NA
< 1 year	NA	6 (6)		NA	24 (2)		NA	5 (11)		NA	19 (8)	
1–5 years	NA	53 (49)		NA	386 (39)		NA	31 (66)		NA	160 (71)	
> 5 years	NA	50 (46)		NA	585 (59)		NA	11 (23)		NA	46 (20)	
Morphology primary tumor												
Ductal carcinoma	91 (90)	96 (88)	0.22	434 (76)	743 (75)	0.12	81 (91)	45 (96)	0.49	86 (91)	186 (83)	0.07
Lobular carcinoma	7 (7)	8 (7)		119 (21)	195 (20)		8 (9)	2 (4)		3 (3)	19 (8)	
Mixed	1 (1)	5 (5)		11 (2)	40 (4)		0 (0)	0 (0)		2 (2)	2 (1)	
Other	2 (2)	0 (0)		7 (1)	17 (2)		0 (0)	0 (0)		3 (3)	18 (8)	
Differentiation grade primary tumor												
Low grade	2 (3)	2 (2)	0.78	55 (12)	127 (15)	0.04	0 (0)	1 (2)	0.25	1 (1)	6 (3)	0.65
Intermediate grade	38 (49)	51 (55)		295 (67)	515 (60)		26 (41)	13 (30)		21 (30)	48 (25)	
High grade	37 (48)	40 (43)		92 (21)	222 (26)		38 (59)	29 (67)		49 (69)	140 (72)	
Unknown	24	16		129	131		25	4		23	30	
Localization of metastases^c^												
Lymph nodes only	8 (8)	2 (2)	0.05	20 (4)	31 (3)	0.67	6 (7)	0 (0)	0.09	10 (11)	13 (6)	0.13
Bone only	28 (28)	25 (23)	0.42	230 (40)	281 (28)	< 0.001	14 (16)	8 (17)	0.84	17 (18)	16 (7)	0.003
Liver	38 (37)	36 (33)	0.48	104 (18)	267 (27)	< 0.001	46 (52)	13 (28)	0.007	31 (33)	75 (33)	0.95
CNS	3 (3)	17 (16)	0.002	15 (3)	35 (4)	0.33	5 (6)	7 (15)	0.07	5 (5)	44 (20)	0.001
Lung	26 (26)	30 (28)	0.77	112 (20)	259 (26)	0.004	21 (24)	11 (23)	0.98	26 (28)	89 (40)	0.04
Other localizations	17 (17)	32 (29)	0.03	141 (25)	347 (35)	< 0.001	16 (18)	14 (30)	0.11	23 (25)	96 (43)	0.002

^a^ Clinical subtype was based on the receptor status of the primary tumor for patients with de novo metastatic disease, and on the receptor status of the metachronous metastasis for those with metachronous metastatic disease. In case the receptor status of the metachronous metastasis was unknown, clinical subtype was based on the receptor status of the primary tumor when available

^b^ P-values were calculated over known values. P-values were calculated using chi2 tests if all cell counts were ≥ 5 and using Fisher’s exact test when any cell count was < 5

^c^ Percentages do not add up to 100% because patients can have metastases at multiple localizations

### Treatment patterns

The proportion of patients receiving any systemic treatment in the metastatic setting was comparable between patients with de novo and metachronous MBC (Table 3). Systemic treatment of patients with HER2-positive tumors more often included chemotherapy (HR+ / HER2+: 66% vs. 53% *p* = 0.04, HR- / HER2+: 78% vs. 55% *p* = 0.007) and/or targeted therapy (HR+ / HER2+: 75% vs. 62% *p* = 0.03) in de novo versus metachronous MBC. In patients with HR+/HER2- tumors, targeted therapy was less frequently given in de novo MBC compared to metachronous MBC (19% vs. 31%, *p* < 0.001). In patients with HR-/HER2- tumors, treatment patterns were similar between de novo MBC and metachronous MBC.

**Table 3:** Systemic treatment in de novo versus metachronous MBC stratified by clinical subtype

	HR+ / HER2+		*p*-value	HR+ / HER2-		*p*-value
	De novo MBC	Metachronous MBC		De novo MBC	Metachronous MBC	
	*N*(%)	*N* (%)		*N*(%)	*N* (%)	
N	101	109		571	995	
**Treatment in metastatic setting**						
Any systemic treatment	84 (83)	82 (75)	0.16	460 (81)	824 (83)	0.26
Systemic treatment included:						
Chemotherapy	67 (66)	58 (53)	0.04	152 (27)	312 (31)	0.05
Targeted therapy	76 (75)	67 (62)	0.03	118 (21)	319 (32)	< 0.001
Hormonal therapy	32 (32)	39 (36)	0.53	378 (66)	647 (65)	0.63
	**HR- / HER2+**			**HR- / HER2-**		
N	89	47		94	225	
**Treatment in metastatic setting**						
Any systemic treatment	71 (80)	34 (72)	0.33	61 (65)	142 (63)	0.76
Systemic treatment included:						
Chemotherapy	69 (78)	26 (55)	0.007	59 (63)	133 (59)	0.54
Targeted therapy	68 (76)	30 (64)	0.12	16 (17)	33 (15)	0.32
Hormonal therapy	0 (0)	4 (8)	0.005	1 (1)	12 (5)	0.08

### Survival

**Fig. 1:**
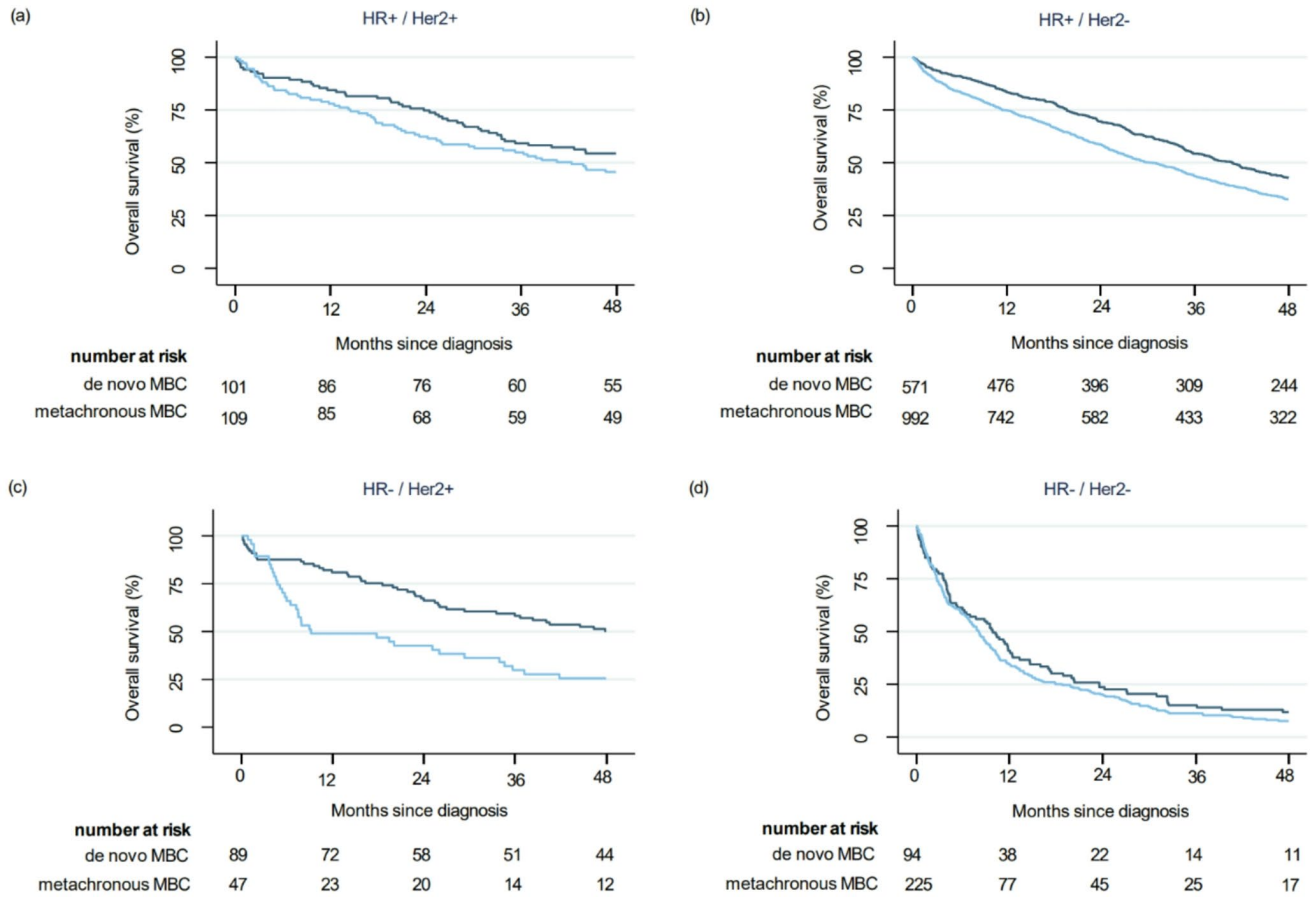
Kaplan-Meier curves of overall survival in de novo versus metachronous MBC stratified by clinical subtype. (**a**) HR+ / HER2+ (**b**) HR+ / HER2-; (**c**) HR- / HER2+; (**d**) HR- / HER2-

Statistically significant better overall survival in de novo MBC was observed in patients with HR+/HER2- and HR-/HER2 + tumors, but not in patients with HR+/HER2 + and HR-/HER2- tumors (Fig. 1/Table 4). The magnitude of differences in overall survival between de novo and metachronous MBC was largest in patients with HR-/HER2 + tumors (51.1 vs. 9.1 months, adjusted HR 1.62 (95%CI 1.03–2.54, *p* = 0.03).

**Table 4:** Median overall survival and Hazard ratios in patients with de novo versus metachronous MBC

	Median OS (months)^a^	95% CI	Hazard ratio (unadjusted)	*P*-value	Hazard ratio (adjusted^b^)	*P*-value
**HR+ / HER2+**						
de novo MBC (*n* = 103)	55.7	35.2 - NA	Reference	0.09	Reference	0.61
metachronous MBC (*n* = 109)	42.3	25.8–55.2	1.35 (0.95–1.92)		1.10 (0.75–1.61)	
**HR+ / HER2-**						
de novo MBC (*n* = 570)	40.8	36.5–44.2	Reference	< 0.001	Reference	< 0.001
metachronous MBC (*n* = 992)	30.3	27.4–33.7	1.33 (1.18–1.50)		1.27 (1.12–1.43)	
**HR- / HER2+**						
de novo MBC(*n* = 89)	51.1	29.3–62.2	Reference	0.006	Reference	0.033
metachronous MBC (*n* = 47)	9.1	6.4–29.3	1.80 (1.18–2.74)		1.62 (1.03–2.54)	
**HR- / HER2-**						
de novo MBC (*n* = 93)	9.7	6.1–12.0	Reference	0.13	Reference	0.69
metachronous MBC (*n* = 225)	8.0	6.6–9.4	1.21 (0.93–1.55)		1.06 (0.80–1.38)	

^a^ Overall survival was assessed from primary diagnosis (de novo metastatic disease) or date of first metastasis (metachronous metastatic disease) until death or end of follow-up

^b^hazard ratios adjusted for age at diagnosis of metastasis, localizations of metastases (lymph node only, bone only, liver, CNS, lung, other), and receipt of systemic treatment in the metastatic setting (yes/no)

Among all patients with de novo or metachronous HR+/HER2 − tumors, 1,276 received systemic therapy (460 de novo; 816 metachronous). Of those patients with metachronous disease, 400 had received prior chemotherapy, 235 prior hormone therapy only, and 181 no prior systemic treatment. Patients without prior systemic treatment had more often stage 1 primary tumors, and their primary tumor was slightly more often diagnosed in earlier years (supplementary Table 3).

**Fig. 2:**
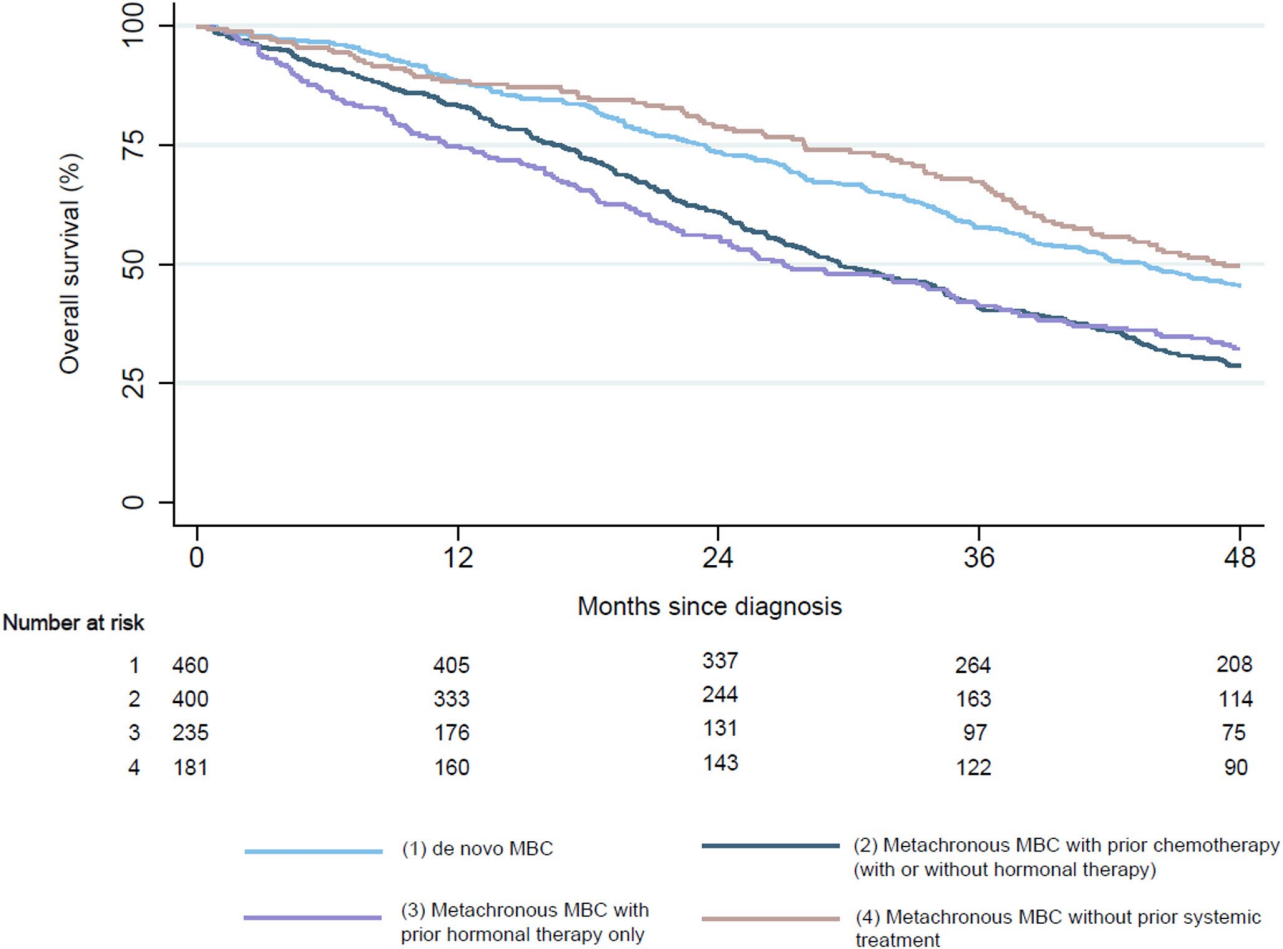
Kaplan-Meier curves for overall survival in de novo versus metachronous MBC in patients with HR+/HER2- tumors who received systemic treatment, stratified by receipt of prior (neo)adjuvant systemic treatment

In patients with HR+/HER2- tumors, patients with metachronous MBC who received prior systemic treatment in the non-metastatic setting had worse overall survival compared to patients with de novo MBC (Fig. 2/Table 5). This was observed both in patients who received prior hormonal therapy and/or chemotherapy (29.6 months vs. 43.6 months, adjusted HR 1.52, *p* < 0.001) and in patients who received prior hormonal therapy alone (27.0 months vs. 43.6 months, adjusted HR 1.33, *p* = 0.003). Patients with metachronous MBC who had not received prior systemic treatment had slightly better overall survival compared to patients with de novo MBC (median survival 47.3 months vs. 43.6 months, adjusted HR 0.79, *p* = 0.03).

**Table 5:** Hazard ratios in de novo versus metachronous MBC in patients with HR+/HER2- tumors who received systemic treatment, stratified by receipt of prior (neo)adjuvant systemic treatment

	Median OS (months) ^a^	95% CI	Hazard ratio (unadjusted)	*P*-value	Hazard ratio (adjusted^b^)	*P*-value
de novo MBC (*n* = 462)	43.6	38.2–46.4	Reference		Reference	
metachronous MBC with prior chemotherapy (with or without hormonal therapy) (*n* = 401)	29.6	27.5–34.3	1.56 (1.33–1.82)	< 0.001	1.52 (1.29–1.78)	< 0.001
metachronous MBC with prior hormonal therapy only (*n* = 234)	27.0	21.8–32.0	1.51 (1.26–1.81)	< 0.001	1.33 (1.10–1.61)	0.003
metachronous MBC without prior systemic treatment (*n* = 176)	47.3	40.3–54.0	0.89 (0.72–1.10)	0.31	0.79 (0.64–0.98)	0.03

^a^ Overall survival was assessed from primary diagnosis (de novo metastatic disease) or date of first metastasis (metachronous metastatic disease) until death or end of follow-up

^b^hazard ratios adjusted for age at diagnosis of metastases and localizations of metastases (lymph node only, bone only, liver, CNS, lung, other)

## Discussion

This population-based study compared clinicopathological characteristics, treatment, and survival between patients with de novo and metachronous MBC diagnosed in the Netherlands in 2019 on a nationwide scale. Our findings demonstrate that these patients present with different characteristics at the time of diagnosis and treatment strategies differ between these two groups of patients. Overall survival was higher in patients with de novo MBC compared to those with metachronous MBC in HR+/HER2- and HR-/HER2 + tumors. However, among patients with HR+ / HER2- metachronous MBC who had not received prior systemic treatment, survival was comparable to that of patients with de novo MBC.

When comparing de novo and metachronous MBC, we observed notable differences in the distribution of clinical subtypes. HER2-positive tumors were more common in de novo MBC compared to metachronous MBC, which may be related to the comprehensive and effective systemic treatment options that have emerged for this subtype, leading to more effective disease control and lower rates of recurrent metastatic disease. This hypothesis is supported by a study showing that recurrence risk has declined most significantly in recent years for HER2-positive tumors [11]. Additionally, a decreasing proportion of HER2-positive recurrent MBC over time has been reported, further supporting this hypothesis [3]. Metastasis-free interval also varied by clinical subtype, with longer metastasis-free intervals in HR+ positive metachronous MBC compared to patients with HR- tumors, aligning with the known association between ER-positive tumors and late recurrence [5, 11–13].

Beyond differences in subtype distribution, our stratified analyses revealed that metastatic patterns also differ within subtypes. Across subtypes, patients with metachronous MBC more frequently presented with central nervous system involvement and a broader range of metastatic sites, including locations beyond the commonly affected lymph nodes, bone, visceral organs, or CNS. Additionally, bone only metastases were less common in metachronous MBC, particularly in HER2- subtypes. Previous studies have reported differences in metastatic patterns between de novo and metachronous MBC [3, 6, 14], however these studies did not stratify by clinical subtype. By accounting for subtype, our findings demonstrate that the observed differences in metastatic patterns are not solely due to subtype distribution, but also reflect distinct disease behavior within subtypes. This suggests that metachronous MBC may be biologically distinct and associated with more unfavorable characteristics [15, 16], even within the same clinical subtype.

Regarding survival outcomes, our study showed the largest survival differences between de novo and metachronous MBC in HR−/HER2 + tumors, while HR-/HER2- tumors showed the smallest differences, aligning with earlier findings [17]. These disparities may partly reflect less favorable disease biology in metachronous MBC, such as differences in metastatic patterns. However, after adjusting for metastatic patterns, survival differences remained significant. Prior (neo)adjuvant treatment exposure may also play a role. This prior treatment could influence both eligibility for and effectiveness of systemic treatment in the metastatic setting due to acquired resistance or lingering side effects, ultimately leading to poorer survival. Our study provides some evidence supporting this explanation. In patients with HR-/HER2 + tumors, where the largest survival difference was observed, patients with metachronous MBC received chemotherapy and targeted therapy at notably lower rates than those with de novo MBC. This may reflect treatment constraints due to prior exposure to anti-HER2 therapy, which may limit treatment options in the metachronous metastatic setting. Interestingly, lower rates of chemotherapy and targeted therapy were also observed in HR+/HER2 + metachronous MBC, yet survival differences in this group were less pronounced. This may be due to the availability of additional endocrine-based treatment options in HR+ disease, which might partially mitigate the impact of reduced chemotherapy or targeted therapy. Additionally, a sub-analysis of patients with HR+/HER2- tumors who received systemic therapy for metastatic disease revealed that those with metachronous MBC who had not received prior systemic treatment had slightly better survival than patients with de novo MBC, after adjusting for age and metastatic patterns. This finding further supports the hypothesis that prior treatment may be associated with survival outcomes in the metachronous metastatic setting. However, it is important to recognize that prior treatment is closely linked to primary tumor stage and may therefore also function as a marker of higher‑risk primary disease. Higher risk disease could itself contribute to poorer survival outcomes, making it difficult to determine how much of the association is attributable to prior treatment versus primary tumor stage. This may become increasingly challenging as the use of (neo) adjuvant treatments is increasing [2, 18], potentially shifting the profile of future metachronous breast cancers toward more therapy-resistant disease [19, 20]. At the same time, there is growing interest in de-escalating (neo)adjuvant treatment, aiming to reduce treatment burden without compromising efficacy [21–23]. In light of our findings, this may not be only beneficial at that time, but may also have favorable implications for treatment options and outcomes in the event of recurrent metastatic disease.

The results of this study emphasize that patients with de novo and metachronous MBC differ not only in subtype distribution but also in disease behavior within biologically similar subgroups. De novo MBC was overall associated with more favorable outcomes. This may support clinicians in reassuring patients, as some may intuitively perceive de novo metastatic disease as having a poorer prognosis compared with recurrence after early‑stage disease. Importantly, the findings of this study also suggest that prior (neo)adjuvant treatment exposure may contribute to less favorable outcomes in metachronous MBC. However, we were only able to explore this in detail within one clinical subtype, highlighting the need for further research to better understand the relationship between prior treatment, resistance mechanisms, and survival in de novo vs. metachronous MBC. Lastly, the results of this study underscore the importance of routine registration of metachronous metastases in cancer registries. This is essential for monitoring and understanding differences in patient characteristics, treatment patterns, and outcomes in clinical practice. In addition, routine reporting and registration of metachronous metastases will support future research on the impact of novel (neo)adjuvant therapies on the characteristics and outcomes of metachronous MBC.

### Strengths and limitations

The main strength of this study is the use of data on both de novo and metachronous metastases on a nationwide basis, which makes it representative of the patient population seen in daily clinical practice. In addition, metastatic disease in the patients in this study was all diagnosed in the same year, allowing an accurate comparison of treatment strategies and prognosis. However, some limitations should be noted. Most importantly, some diagnoses of metachronous metastases may be missed due to the methods of identification. Metachronous metastases were identified based on the pathology archive, an algorithm using hospital data, and/or manual follow-up by data managers. Because the first two methods rely on whether a patient received pathologic confirmation or whether a patient received hospital care, those patients without pathologic confirmation of metachronous metastases and with limited hospital contact (i.e., patients who declined extensive diagnostics and/or treatment) may have been more difficult to identify and may have resulted in underreporting of metachronous metastases in this study. Second, the use of systemic treatments in the metastatic setting, as reported in this study based on hospital data from the LBZ, is subject to underreporting. A previous Dutch study reported higher rates of systemic treatment use among patients with metastatic MBC [6]. Furthermore, when we supplemented the LBZ data with information on initial treatment for de novo MBC from the NCR we observed higher rates of systemic treatment use, particularly for hormonal therapies (Supplementary Table 4). This incompleteness might relate to the fact that in the Netherlands hormonal therapy is given outside the hospital in general pharmacies of which the data is not part of the LBZ. Additionally, hormonal therapy is not included among the dispensed medications in the LBZ, but is instead recorded only as medical procedures related to the administration of or guidance by hormonal therapy. As a result, the use of hormonal therapy may be less prominently reflected in the LBZ compared to chemotherapy and targeted therapies, which are directly registered as dispensed medications. However, as this underreporting is expected to be similar in de novo and metachronous MBC, it is not expected to affect the comparisons made in this study. Lastly, it is important to note that some subgroups had relatively small sample sizes (e.g., 47 patients in the HR- / HER2 + metachronous MBC group), which limits the precision of the survival estimates and results in relatively wide confidence intervals. These findings should therefore be interpreted with caution.

## Conclusion

Patients with de novo and metachronous metastatic breast cancer represent two distinct clinical groups at the time of metastatic diagnosis, differing not only in subtype distribution but also in disease characteristics and survival within biologically similar subgroups. Our findings suggest that prior systemic treatment in metachronous MBC may contribute to these differences, as patients without prior treatment had outcomes comparable to those with de novo MBC. These findings highlight the importance of further research to better understand the relationship between prior treatment and survival, as well as the routine registration of metachronous metastases in cancer registries.

## Electronic Supplementary Material

Below is the link to the electronic supplementary material.

Supplementary Material 1

## Data Availability

The datasets analyzed during the current study are not publicly available due to confidentiality but are available from the Netherlands Cancer Registry on reasonable request and subject to approval. Requests can be made through the application form which can be found at: https://iknl.nl/en/ncr/apply-for-data. Further information is available from the corresponding author upon request.
