# Supplementary material for: Characteristics, treatment and survival in de novo and metachronous metastatic breast cancer: a nationwide comparative analysis
Source: Breast Cancer Res Treat. 2026 May 12;217(2):33. doi: 10.1007/s10549-026-07979-5 (PMC13167865; doi:10.1007/s10549-026-07979-5)
Supplement: Supplementary file 1 — Supplementary Material 1 [file 10549_2026_7979_MOESM1_ESM.docx]

# Supplementary material

*Supplementary table 1: Clinical subtype in metachronous metastatic breast cancer before and after substitution of unknowns with clinical subtype of the primary tumor.*

| **Clinical subtype** | **De novo MBC**  **N (%)** | **Metachronous MBC – subtype at time of metastasis**  **N (%)** | **Metachronous MBC - with unknowns substituted based on subtype primary tumor**  **N (%)** |
| --- | --- | --- | --- |
| HR+ / Her2+ | 103 (11) | 75 (5) | 109 (7) |
| HR+ / Her2- | 570 (63) | 579 (40) | 995 (68) |
| HR- / Her2+ | 89 (10) | 28 (2) | 47 (3) |
| HR- / Her2- | 93 (10) | 118 (8) | 225 (15) |
| Unknown/undetermined | 45 (5) | 666 (45) | 90 (6) |

*Supplementary table 2: Concordance between subtype of the primary tumor and subtype of metachronous metastases in patients in whom both subtypes were known (n=672).*

|  | **Subtype at time of metachronous metastases** | | | |
| --- | --- | --- | --- | --- |
| **Subtype primary tumor** | HR+ / Her2+ | HR+ / Her2- | HR- / Her2+ | HR- / Her2- |
| HR+ / Her2+ (n=58) | 41 (71) | 9 (16) | 4 (7) | 4 (7) |
| HR+ / Her2- (n=510) | 19 (4) | 444 (87) | 2 (0) | 45 (9) |
| HR- / Her2+ (n=34) | 9 (26) | 3 (9) | 18 (53) | 4 (12) |
| HR- / Her2- (n=70) | 0 (0) | 12 (17) | 0 (0) | 58 (83) |

*Supplementary table 3: Characteristics of patients with HR+/Her2 metachronous MBC who received systemic treatment, stratified by receipt of prior (neo)adjuvant systemic treatment*

|  | **Prior chemotherapy (with or without hormonal therapy)** | **Prior hormonal therapy only** | **No prior systemic treatment** |
| --- | --- | --- | --- |
|  | 400 | 235 | 181 |
| Primary tumor stage |  |  |  |
| I | 22 (6) | 51 (22) | 141 (78) |
| II | 223 (56) | 144 (61) | 33 (18) |
| III | 155 (39) | 39 (17) | 3 (2) |
| Unknown | 0 (0) | 1 (0) | 4 (2) |
| Age at primary tumor diagnosis |  |  |  |
| <50 | 167 (42) | 16 (7) | 24 (13) |
| 50-70 | 229 (57) | 117 (50) | 122 (67) |
| 70+ | 4 (1) | 102 (43) | 35 (19) |
| Initial diagnosis year |  |  |  |
| <2000 | 9 (2) | 6 (3) | 14 (8) |
| 2000-2004 | 31 (8) | 13 (6) | 19 (11) |
| 2005-2009 | 88 (22) | 30 (13) | 42 (23) |
| 2010-2014 | 141 (35) | 79 (34) | 48 (27) |
| 2015-2019 | 131 (33) | 107 (46) | 58 (32) |

*Supplementary table 4: Systemic treatment in de novo MBC based on data from the Dutch National Hospital Care Registration (LBZ) alone and supplemented with data on initial treatment from the Netherlands Cancer Registry (NCR).*

|  | **HR+ / HER2+** | | **HR+ / HER2-** | | **HR- / HER2+** | | **HR- / HER2-** | |
| --- | --- | --- | --- | --- | --- | --- | --- | --- |
|  | **LBZ** | **LBZ and NCR** | **LBZ** | **LBZ and NCR** | **LBZ** | **LBZ and NCR** | **LBZ** | **LBZ and NCR** |
|  | **N (%)** | **N (%)** | **N (%)** | **N (%)** | **N (%)** | **N (%)** | **N (%)** | **N (%)** |
| N | 101 | 101 | 571 | 571 | 89 | 89 | 94 | 94 |
| **Treatment in metastatic setting** |  |  |  |  |  |  |  |  |
| Any systemic treatment | 84 (83) | 96 (96) | 460 (81) | 547 (96) | 71 (80) | 80 (90) | 61 (65) | 69 (73) |
| Systemic treatment included: |  |  |  |  |  |  |  |  |
| *Chemotherapy* | 67 (66) | 78 (77) | 152 (27) | 169 (30) | 69 (78) | 79 (89) | 59 (63) | 68 (72) |
| *Targeted therapy* | 76 (75) | 87 (86) | 118 (21) | 131 (23) | 68 (76) | 79 (89) | 16 (17) | 18 (19) |
| *Hormonal therapy* | 32 (32) | 74 (73) | 378 (66) | 527 (93) | 0 (0) | 0 (0) | 1 (1) | 4 (4) |
